# Comparison of CK-IHC assay on serial frozen sections, the OSNA assay, and in combination for intraoperative evaluation of SLN metastases in breast cancer

**DOI:** 10.1007/s12282-017-0811-y

**Published:** 2017-11-01

**Authors:** Hideo Shigematsu, Shinji Ozaki, Daisuke Yasui, Junichi Zaitsu, Daiki Taniyama, Akihisa Saitou, Kazuya Kuraoka, Hiroyasu Yamashiro, Kiyomi Taniyama

**Affiliations:** 1 Department of Breast Surgery, National Hospital Organization Kure Medical Center and Chugoku Cancer Center, 3-1, Aoyama-cho, Kure, Hiroshima 737-0023 Japan; 2 Department of Pathology, National Hospital Organization Kure Medical Center and Chugoku Cancer Center, Kure, Hiroshima Japan; 3 Department of Breast Surgery, Tenri Hospital, Nara, Nara Japan; 4 National Hospital Organization Kure Medical Center and Chugoku Cancer Center, Kure, Hiroshima Japan

**Keywords:** Breast cancer, Intraoperative diagnosis, SLN, OSNA, CK-IHC

## Abstract

**Background:**

Intraoperative evaluations of sentinel lymph node (SLN) metastases are performed for providing appropriate and immediate axillary treatments in breast cancer patients who do not meet Z0011 criteria; however, standard intraoperative procedure has not yet been established.

**Methods:**

We consecutively performed intraoperative evaluation for SLN metastases using both a cytokeratin immunohistochemistry (CK-IHC) assay on serial frozen sections and a one-step nucleic acid amplification (OSNA) assay of the remaining whole node in patients with invasive breast cancer. In this article, we compared the intraoperative diagnostic ability of CK-IHC assay, the OSNA assay, and in combination.

**Results:**

Between August 2009 and May 2017, 1,103 SLNs from 499 consecutive clinically node-negative invasive breast cancers were intraoperatively evaluated for SLN metastases using an OSNA and CK-IHC assay. The detection rates of SLN metastases by the OSNA and CK-IHC assays and in combination were 11.8, 12.1, and 14.5%, respectively. The concordance rate between the intraoperative SLN findings of the OSNA and CK-IHC assays was 94.9% (95% confidence interval 93.6–96.2%). The false negative rate for the OSNA assay was 3.1% (30/973), including 3 (0.3%) macrometastases and 27 (2.8%) micrometastases, and for the CK-IHC assay was 2.7% (26/969), including 1 (0.1%) OSNA ++ and 25 (2.6%) OSNA +.

**Conclusions:**

The CK-IHC assay and the OSNA assay have compatible diagnostic abilities in intraoperative evaluations for SLN metastases. The low incidence of false negative results with limited disease burden suggests that both assays can be reliable techniques for intraoperative diagnoses of SLN metastases in breast cancer patients.

## Introduction

Sentinel lymph node (SLN) biopsy is the standard procedure for patients with clinically node-negative breast cancer [1]. Although axillary lymph node dissection (ALNDs) have been performed for patients with involved SLN, there has been a marked decrease in the frequency of ALND since the publication of the American College of Surgeons Oncology Group (ACOSOG) Z0011 study [2–4]. Updated American Society of Clinical Oncology clinical guidelines recommend the omission of ALND for involved SLN in breast cancer patients who meet Z0011 criteria; clinical T1–T2 invasive breast cancer, no palpable adenopathy, 1–2 SLNs containing metastases identified by frozen section, touch preparation, or hematoxylin–eosin staining on permanent section, treatment with lumpectomy, no neoadjuvant treatment and no gross extranodal disease [1]. However, ALND is still the standard of care for breast cancer patients with SLN involvement that undergo mastectomy or have received neoadjuvant chemotherapy, which are exclusion criteria of Z0011. The intraoperative diagnosis of SLN metastases remains important in these patients for providing appropriate and immediate axillary surgery without delay.

Although several techniques have been proposed for intraoperative evaluation of SLN metastases, no standard intraoperative procedure has been established. Intraoperative hematoxylin and eosin (H&E) pathological examinations of frozen section, imprint cytology or cell smear are associated with substantial false negative results in comparison with postoperative evaluations on permanent sections and the average false negative rate of these intraoperative techniques is 25% in cases with a positive node [5–7]. The incorporation of a rapid cytokeratin immunostaining technique into cytological or histological evaluation was reported to improve diagnostic ability with results comparable to those of permanent pathological examination [5, 8, 9], however, the data are limited. The one-step nucleic acid amplification (OSNA) assay has been developed as a molecular method that uses the reverse transcriptase polymerase chain reaction of cytokeratin (CK)-19 mRNA for the diagnoses of SLN metastases [10–16]. The OSNA assay can allow intraoperative evaluation of entire LNs, which overcome problems of sampling limitations and showed equivalent diagnostic ability for detection of SLN metastases compared with conventional pathological assay. However, the destructive process to lymph node tissue disturbs the measurement of the disease burden and morphologic evaluation is a concern.

Considering the above-mentioned limitations of each intraoperative technique, we consecutively performed intraoperative evaluation for SLN metastases using both a cytokeratin immunohistochemistry (CK-IHC) assay on serial frozen sections cut at 2 mm intervals and a one-step nucleic acid amplification (OSNA) assay of the remaining whole node in patients with invasive breast cancer. In this article, we compared the CK-IHC assay, the OSNA assay, and in combination for intraoperative evaluations of SLN metastases in breast cancer.

## Materials and methods

### Patients and methods

A total of 1,103 SLNs from 499 consecutive, clinically node-negative invasive breast cancers (cT1-T3N0M0) were intraoperatively evaluated for the presence of SLN metastases, using both the OSNA and CK-IHC assays, between August 2009 and May 2017 at the Kure Medical Center and the Chugoku Cancer Center, Kure, Japan. Clinical node-negativity was determined by physical examinations and imaging techniques, including ultrasonography, computed tomography, or magnetic resonance imaging. Fine-needle aspirations or core needle biopsies were performed to confirm the presence of metastases in regional lymph nodes when ultrasound showed normal-sized or enlarged lymph nodes with irregular morphological structure of hilum or thickening of cortex. This study was approved by the Kure Medical Center review board (28–78), which waived the requirement for informed consent from individual patients because this study was a retrospective review of a prospectively maintained patient database.

### Clinicopathological factors

The preoperative clinicopathologic variables in this study were derived from a prospectively maintained database at our institute, and included patient age, histological type of cancer, prescribed neoadjuvant therapy, clinical T stage according to TNM classification, nuclear grade, estrogen receptor (ER) status, progesterone receptor (PgR) status, human epidermal growth factor receptor 2 (HER2) status and type of breast surgery (Table 1). ER and PgR status were determined using immunohistochemistry (IHC) assays, and tumors with 1% or more of positively-stained tumor cells that were classified as positive for ER and PgR. The HER2 status of the tumors were determined using IHC and/or fluorescence in situ hybridization (FISH) analysis; HER2-positive tumors were defined according to an IHC scoring of 3 + or HER2 gene amplification using FISH [17].

**Table 1:** Patients’ characteristics

Factor	Number of cases	%
Total	499	
Age	Median 64 (range 26–93)	
Histological subtype		
Invasive ductal carcinoma	435	87.2
Invasive lobular carcinoma	25	5.0
Mucinous carcinoma	25	5.0
Special type	14	3.0
Neoadjuvant therapy		
Chemotherapy	49	9.8
Endocrine therapy	6	1.2
No	444	89.0
Clinical T stage		
T1	333	66.7
T2	154	30.9
T3	12	2.4
Nuclear grade		
1	327	65.5
2	104	20.8
3	68	13.6
ER status		
Positive	435	87.2
Negative	64	12.8
PgR status		
Positive	372	74.5
Negative	127	25.5
HER2 status		
Positive	73	14.6
Negative	422	84.6
Unknown	4	0.8
Breast surgery		
Breast-conserving surgery	325	65.1
Mastectomy	174	34.9

*ER* estrogen receptor, *PgR* progesterone receptor, *HER2* human epidermal growth factor receptor 2

### SLN biopsy and evaluation procedure

**Fig. 1:**
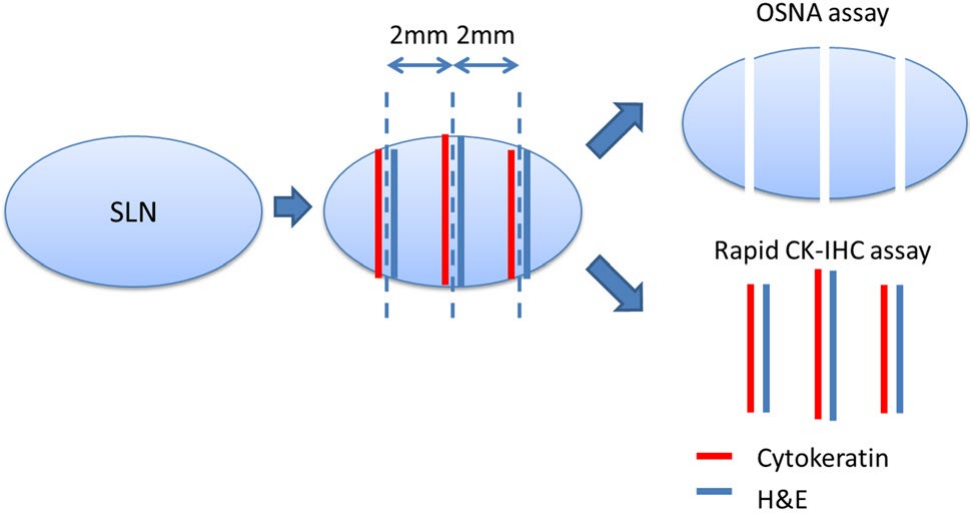
Preparation of sentinel lymph nodes for the OSNA and CK-IHC assay. Surgically obtained axillary lymph nodes were cut at 2 mm or less intervals. The paired 4-μm thick sections of each piece were intraoperatively examined with H&E and rapid IHC staining for cytokeratin. All remaining specimens were subjected for OSNA assay to detect sentinel lymph node metastases

SLN biopsies were performed using both dye colloid and radioactive colloid (technetium-99m phytate). On the day before surgery, radioactive colloid was injected into the subdermal space of the areola and lymphoscintigraphy was performed 2 h after this injection to identify the location of SLNs. SLN biopsy was performed at the same time as the primary breast tumor resection. Five mg (1 mL) of indocyanine green, 3 mL of indigo carmine, or both, were subdermally injected in the areola, and the SLNs were identified using a gamma probe and dye mapping [18, 19]. Lymph nodes with dye uptake, radiotracer uptake, or both, were classified as SLNs. Subjected SLNs were intraoperatively evaluated for metastases using both the OSNA and CK-IHC assays (Fig. 1). The frozen SLNs were cut at intervals of 2 mm or less, and paired 4-μm-thick sections of each piece were intraoperatively examined with H&E staining and CK-IHC staining for cytokeratin as shown previously [20]. Briefly, the sliced tissues were fixed for 30 s in liquid nitrogen, washed in saline, and rinsed in Tris-buffered saline (TBS). For CK-IHC, the anti-cytokeratin antibody (Cytokeratin, Wide Spectrum Screening, DAKO) was used as the primary antibody, and labeled polymer-HRP as a secondary antibody (Histofine simple stain MAX-PO, Nichirei). H&E staining was performed, and the histologic findings were compared with those of IHC staining. Distinct staining of the cells was considered as positive staining for cytokeratin. The entire procedure was essentially completed within 30 min. Isolated or no tumor cells were recognized as negative, and micrometastases or macrometastases were recognized as positive, according to the criteria of the Seventh Edition of the Tumor-Node-Metastases classification of the Union for International Cancer Control. The OSNA assay was performed as previously described to detect SLN metastases in the all remaining specimens (Fig. 1). SLNs were classified as OSNA − (CK19 mRNA < 2.5 × 10^2^ copies/μL), OSNA + (2.5 × 10^2^ to < 5.0 × 10^3^ copies/μL), and OSNA ++ (≥ 5.0 × 10^3^ copies/μL) [10, 21]. In this study, OSNA + and OSNA ++ SLNs were regarded as positive for SLN metastases. When a presence of heterotopic epithelia was recognized in SLN by histopathological assay, the SLN was omitted from the OSNA assay due to concerns of false positive results.

### Statistical analysis

The SLN metastases detection rate of OSNA assay, CK-IHC assay, or in combination was compared using the *χ* ^2^ test. The concordance rates in detection of SLN metastases and measurement of disease burden in involved SLNs between OSNA and CK-IHC assay were evaluated. Statistical analyses were carried out using SPSS software (version 11 for Windows; SAS Institute, Tokyo, Japan). A *p* value of < 0.05 was considered as statistically significant.

## Results

### Intraoperative detection rates of SLN metastases by OSNA, CK-IHC assay, and in combination

Of the 1,103 SLNs of 499 cases evaluated in this study, the detection rates of SLN metastases by the OSNA and CK-IHC assays and in combination were 11.8, 12.1, and 14.5%, respectively (Table 2), showing that the combination assay detected marginally more SLN metastases than the OSNA assay (*p* = 0.059) or the CK-IHC assay (*p* = 0.10), individually. Of the 130 OSNA-positive SLNs, 68 (52.3%) were OSNA ++, and 62 (47.7%), OSNA +. Of the 134 SLNs analyzed by the CK-IHC assay, 74 (55.2%) were macrometastases, and 60 (44.8%), micrometastases.

**Table 2:** Detection rate of SLN metastases by intraoperative CK-IHC assay, OSNA assay and combination assay in (A) whole, (B) mastectomy, (C) neoadjuvant and (D) T3 population

Intraoperative procedure	SLN metastasis			*p* value*
	Positive	Negative	%	
A. Whole population				
CK-IHC and OSNA assay	160	943	14.5	
Rapid CK-IHC alone	134	969	12.1	0.10
OSNA alone	130	973	11.8	0.059
B. Mastectomy population				
CK-IHC and OSNA assay	90	317	22.1	
Rapid CK-IHC alone	77	330	18.9	0.26
OSNA alone	71	336	17.4	0.095
C. Neoadjuvant population				
CK-IHC and OSNA assay	8	116	6.5	
Rapid CK-IHC alone	8	116	6.5	1.0
OSNA alone	4	120	3.2	0.24
D. T3 population				
CK-IHC and OSNA assay	12	19	38.7	
Rapid CK-IHC alone	12	19	38.7	1.0
OSNA alone	8	23	25.8	0.28

* *p* value was evaluated using *χ* ^2^ test

*ER* estrogen receptor, *PgR* progesterone receptor, *HER2* human epidermal growth factor receptor 2

Because omission of ALND is not recommended in cases with neoadjuvant treatment, mastectomy or T3 tumors in accordance with Z0011 criteria, subgroup analyses based on these factors were performed (Table 2). One hundred and twenty-five SLNs of 55 cases administered with neoadjuvant therapy were evaluated, as were 407 SLNs of 174 cases treated with mastectomy and 31 SLNs of 12 patients with T3 tumor. The incidence of SLN metastases in cases administered with neoadjuvant therapy was significantly lower than in those that were not (6.4 vs. 14.5%, *p* = 0.013) and most of positive SLN (7/8) were micrometastasis. There was no difference in the detection rates of SLNs between the OSNA, CK-IHC, and in combination (3.2 vs. 6.5 vs. 6.5%), however, half of positive SLN were negative for OSNA assay. The incidence of SLN involvement in cases who underwent mastectomies was significantly higher than in cases that underwent breast-conserving surgery (22.1 vs. 10.1%, *p* < 0.001). The detection rates of SLN metastases by the OSNA and CK-IHC assays and in combination were 17.4, 18.9, and 22.1%, respectively, with a tendency toward a higher detection rate in the combination assay. The incidence of SLN involvement in cases with T3 tumor was significantly higher than in cases with T1/T2 tumor (38.7 vs. 13.8%, *p* < 0.001). The detection rates of SLN metastases by the OSNA and CK-IHC assays and in combination were 25.8, 38.7, and 38.7%.

### Correlation between OSNA and CK-IHC assay for intraoperative findings of SLN metastases

**Fig. 2:**
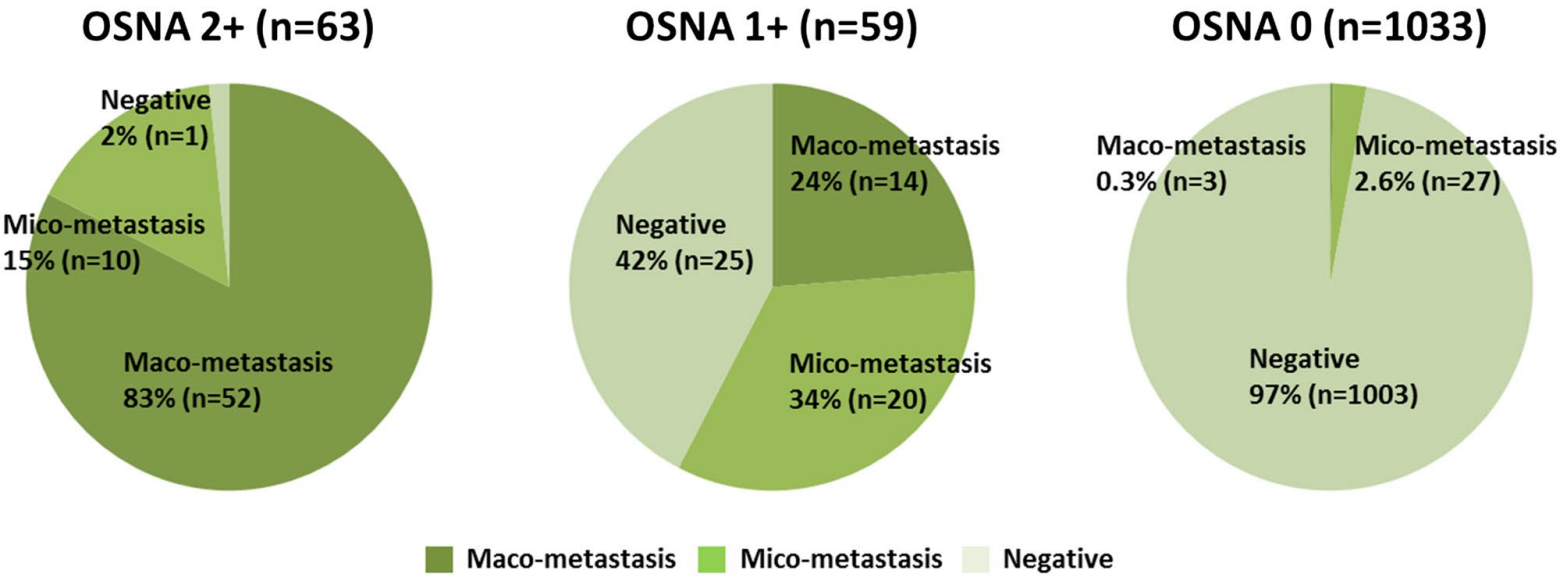
Comparison between OSNA score (OSNA ++, +, 0) and size of pathological metastases (macrometastasis, micrometastasis, negative) by CK-IHC assay

The correlation between findings of the OSNA and of the CK-IHC assay on frozen sections was evaluated (Table 3). In regard to the diagnosis for SLN involvement, the concordance between the OSNA and CK-IHC assays was 94.9% [95% confidence interval (CI) 93.6–96.2%]. Of the 973 SLNs that were negative for metastases per the OSNA assay, 3 (0.3%) had macrometastases, 27 (2.8%) micrometastases, and 943 (96.9%) did not show any pathological metastases, indicating that the false negative rate of the OSNA assay was 3.1% (95% CI 2.0–4.2%). Of the 969 SLNs that were negative per the CK-IHC assay, 1 (0.1%) was OSNA ++, 25 (2.6%) OSNA +, and 943 (97.3%) OSNA −, indicating that the false negative rate of the CK-IHC assay was 2.7% (95% CI 1.7–3.7%). The correlation between the OSNA scores and the sizes of metastases evaluated by the CK-IHC assay were also evaluated (Fig. 2). OSNA ++ SLNs showed macrometastases at a rate of 83.8% (57/68) and micrometastases at 14.7% (10/68). OSNA + SLNs showed 22.6% (14/59) macrometastases and 37.1% (23/59) micrometastases by the CK-IHC assay. The remaining 25 (40.3%) OSNA + SLNs showed no metastases by the CK-IHC assay. Regarding the burden of SLN metastases, the concordance rate between the OSNA scores and the histological size of metastases was 50.0% (95% CI 42.1–57.8%).

**Table 3:** Comparison of intraoperative OSNA and CK-IHC assay in the diagnosis of SLN metastasis

	Rapid CK-IHC			
	Macro-meta	Micro-meta	Negative	Total
OSNA				
2 +	57	10	1	68
1 +	14	23	25	62
0	3	27	943	973
Total	74	60	969	1103

*SLN* sentinel lymph node, *CK-IHC* cytokeratin immunohistochemistry, *OSNA* one-step nucleic acid amplification

## Discussion

In this article, we compared the diagnostic ability of a comprehensive molecular method by OSNA assay with a detailed morphologic method by CK-IHC assay on frozen sections cut at 2 mm intervals, and showed that both assays have equivalent diagnostic ability for intraoperative evaluation of SLN metastases in invasive breast cancer. There were false negative results in both assays; however, the low incidences and limited disease burden of a false negative suggests that both assays can be reliable intraoperative techniques for SLN metastases in breast cancer patients.

The diagnostic ability of the CK-IHC assay on frozen sections in this article seems to be superior to the results in previous studies of intraoperative pathological analysis on a frozen section [6, 7, 22, 23]. The pathological assay using the permanent sections cut at 2-mm interval was shown to have compatible diagnostic ability with OSNA assay [10]. However, the intraoperative diagnostic ability using serial frozen sections seems to inferior to the postoperative one using permanent sections [24]. We think that intraoperative CK-IHC assay in addition to HE analysis has improved the intraoperative diagnostic ability of SLN involvement in this study [25, 26]. One may argue that the procedure of intraoperative CK-IHC assay on multiple surfaces of frozen samples is too laborious for pathologists and medical technologists; however, we have established a system for the intraoperative CK-IHC assay and complete the procedures within 30 min [20].

The OSNA assay is a new molecular method for diagnosis of SLN metastasis and the diagnostic ability of the OSNA assay was reported to be as accurate as pathological assay on permanent section [10, 27, 28]. However, the results of the OSNA assay should be evaluated with some cautions. First, there is no certain concordance between the OSNA scores and the pathological size of metastases. Although a modest correlation was recognized between OSNA ++ and macrometastasis, there was weak correlation between OSNA + and micrometastasis in this study. Because the pathological size of SLN metastases was associated with long-term survival, the prognostic value of the OSNA score should be evaluated in future study [29, 30]. Second, the application of an OSNA assay is controversial in the case of neoadjuvant treatment. A previous study reported that expression of CK19 mRNA may be altered by chemotherapy which leads to a false negative result [31]. The effect of neoadjuvant treatment in the lymph node tissue may be overlooked. The presence of fibrosis or scar tissue in lymph node indicates an eradication of occult metastases by neoadjuvant therapy; however, pathologists cannot evaluate the effect because of the destructive processing in the OSNA assay. The recognition of occult metastases upstages the residual disease which can affect the decision regarding adjuvant therapy [32, 33]. The destructive process also disturbs the morphologic evaluation. For example, the presence of extranodal invasions, which is recognized in approximately a quarter of cases with a positive node and is associated with poor prognosis in breast cancer, cannot be evaluated by the OSNA assay [34, 35].

Although the OSNA and CK-IHC assay in combination detected more SLN metastases compared with either one individually, the incidences of false negative results in both the OSNA and CK-IHC assay were relatively low and most cases were micrometastases or OSNA +. Considering the labor involved in a CK-IHC assay or special equipment and reagent of the OSNA assay, either assay is sufficient for intraoperative diagnosis of SLN metastases in daily practice. However, the combination of the OSNA and CK-IHC assay can be considered in cases with neoadjuvant therapy, mastectomy, or T3 tumors. First, there were high incidences of SLN involvement in cases with mastectomies or T3 tumor [36], which can lead to substantial false negative results in either CK-IHC or OSNA assay alone. Second, OSNA assay has several limitations in case of neoadjuvant treatment as discussed above. The combined assay or pathological assay should be considered instead of OSNA assay alone. Finally, because these factors are the exclusion criteria of Z0011 trial, false negative results of axillary disease should not be ignored to provide appropriate axillary treatment in patients who undergo SLN biopsy.

In conclusion, the OSNA and CK-IHC assays on frozen sections have compatible diagnostic abilities in intraoperative evaluations for SLN metastases. The low incidences of false negative results with a limited disease burden suggest that both assays can be reliable techniques for intraoperative diagnoses of SLN metastases in breast cancer patients. However, the selection of a technique for intraoperative evaluation of SLN metastasis may be determined by the presence of neoadjuvant treatment, mastectomy or T3 tumor.

## Abbreviations

ALND: Axillary lymph node dissection
SLN: Sentinel lymph node
OSNA: One-step nucleic acid amplification assay
CK: Cytokeratin
IHC: Immunohistochemistry
H&E: Hematoxylin and eosin
ER: Estrogen receptor
PgR: Progesterone receptor
HER2: Human epidermal growth factor receptor 2
FISH: Fluorescence in situ hybridization
